# Five-mer peptides prevent short-term spatial memory deficits in Aβ25-35-induced Alzheimer’s model mouse by suppressing Aβ25-35 aggregation and resolving its aggregate form

**DOI:** 10.1186/s13195-023-01229-2

**Published:** 2023-04-19

**Authors:** Rina Nakamura, Motomi Konishi, Youichirou Higashi, Motoaki Saito, Toshifumi Akizawa

**Affiliations:** 1 Department of Pharmacology, Kochi Medical School, Kochi University, Kohasu, Oko-Cho, Nankoku, Kochi 783-8505 Japan; 2 O-Force Co., Ltd, 3454 Irino Kuroshio-Cho, Hata-Gun, Kochi, 789-1931 Japan; 3 Department of Integrative Pharmaceutical Sciences, Faculty of Pharmaceutical Sciences, Setsunan University, 45-1 Nagaotoge-Cho, Hirakata, Osaka 573-0101 Japan

**Keywords:** Alzheimer’s disease, Amyloid β peptide, 5-Mer synthetic peptide, Aggregation, ThT assay

## Abstract

**Background:**

The development of drugs for Alzheimer’s disease (AD), which is related to the misfolding and aggregation of amyloid-β (Aβ), is high in demand due to the growing number of AD patients. In this study, we screened 22 kinds of 5-mer synthetic peptides derived from the Box A region of Tob1 protein to find a peptide effective against Aβ aggregation.

**Methods:**

A Thioflavin T (ThT) assay was performed to evaluate aggregation and screen aggregation inhibitors. Male ICR mice (6 weeks old) were administered saline, 9 nmol Aβ25-35, or a mixture of 9 nmol Aβ25-35 and 9 nmol GSGFK in the right lateral ventricle. Short-term spatial memory was assessed through Y-maze. Microglia cells (BV-)2 cells were plated on 24-well plates (4 × 10^4^ cells/well) and incubated for 48 h, and then, the cells were treated with 0.01, 0.05, 0.1, 0.2, or 0.5 mM GSGFK. After incubation for 24 h, bead uptake was evaluated using a laser confocal microscope and Cytation 5.

**Results:**

We found two kinds of peptides, GSGNR and GSGFK, that were not only suppressed by aggregation of Aβ25-35 but also resolved the aggregated Aβ25-35. Results obtained from the Y-maze test on an Aβ25-35-induced AD model mouse indicated that GSGFK prevents the deficits in short-term memory induced by Aβ25-35. The effect of GSGFK on phagocytosis in BV-2 cells proved that GSGFK activates the phagocytic ability of microglia.

**Conclusions:**

In conclusion, 5-mer peptides prevent short-term memory deficit in Aβ25-35 induced AD model mouse by reducing the aggregated Aβ25-35. They may also upregulate the phagocytic ability of microglia, which makes 5-mer peptides suitable candidates as therapeutic drugs against AD.

## Background

Alzheimer’s disease (AD) is the most common age-related neurodegenerative disorder. Amyloid-β (Aβ) 42, one of the causes of AD, is produced by the cleavage of amyloid precursor protein (APP) by β- or γ-secretases [1, 2]. Since Aβ42 oligomers exhibit strong neurotoxicity, Aβ42 is a potential target for drug therapies [3–7]. Two strategies, involving inhibitors against β- or γ-secretases and against Aβ42 oligomerization, have been followed to develop drugs against AD; however, there are no reports of their effectiveness on established cases of AD.

Recent evidence suggests that in AD patients, Aβ25-35 is produced via the enzymatic cleavage of Aβ42 and that Aβ25-35 can induce alterations in neuronal activity along with damage to long-term memory [8–10]. Furthermore, administration of Aβ25-35 in the CA1 subfield of the rat hippocampus induces morphological changes in the granular cells of the dentate gyrus as well as impairment of memory retrieval [11]. Aβ25-35 intracerebroventricular (i.c.v.) injected mice showed impairment in alternation behaviour in the Y-maze test. Histological examination indicated that i.c.v. injection of Aβ25-35 induced cell loss, and we observed deposition of amyloid in the brain [12]. Therefore, Aβ25-35-induced AD model mouse are suitable for screening AD drugs [13–15]. Several researchers have also carried out conformational studies of Aβ25-35 and toxicity in cells and showed that Aβ25-35 undergoes a conformational change from a soluble to an aggregated β-structure form [16, 17]. Aβ25-35 decreased cell viability by increasing apoptosis and abnormal nuclear morphology [18]. Some researchers revealed that the enhanced content of cholesterol in the cell membrane by Aβ25-35 led to neuronal apoptosis [19]. Thus, the use of Aβ25-35 peptide has contributed considerably toward understanding the effect of Aβ toxicity and aggregation mechanisms.

We recently reported synthetic peptides, JAL-TA9 (YKGSGFRMI), which possess proteolytic activity and cleave Aβ fragment peptides [20, 21]. Our study was the first to report this peptide enzyme. Therefore, we used the term Catalytide (catalytic peptide) for the shorter proteolytic peptides [21]. JAL-TA9 is derived from the Box A region of Tob1, a member of the Tob/BTG family comprising BTG1, BTG2, BTG3/ANA, BTG4, and Tob2 [22–25]. The catalytic centre of JAL-TA9 was identified as GSGFR. Furthermore, a structure–activity relationship study showed that GSGYR, a point mutated peptide of GSGFR, also showed proteolytic activity and cleaved Aβ11-29 [26, 27].

In this study, we evaluated the effects of GSGFR and 21 point-mutated GSGFR peptides against both Aβ25-35 aggregation and its aggregated form. Two kinds of 5-mer peptides, GSGNR and GSGFK, inhibited Aβ25-35 aggregation but did not show proteolytic activity. In addition, to examine whether GSGFK prevents deficits in short-term spatial memory induced by Aβ25-35, we conducted a Y-maze test [28]. As a result, two kinds of 5-mer peptides, GSGNR and GSGFK, were identified as effective candidates for a new AD treatment strategy.

## Methods

### Peptide preparation

The peptides were prepared as described previously [21]. Briefly, the peptides were synthesized using an automated peptide synthesizer (model 433A, Applied Biosystems, CA, USA, 0.1 mmol scale with preloaded resin) and purified using reverse-phase high-performance liquid chromatography. The purified peptide was characterized by electrospray ionization-mass spectrometry using a Qstar Elite Hybrid LC–MS/MS system.

### Thioflavin T-assay

A Thioflavin T (ThT) assay was performed to evaluate aggregation [27]. The amyloid β-peptides (at a final concentration of 100 μM) were incubated with ThT solution (at a final concentration of 100 μM) in Tris–HCl buffer, pH 7.5 or PBS. The ThT signal was monitored by measuring fluorescence emission at 480 nm for 10 s when excited at 444 nm using a Cytation 5 (BioTek). Aggregated Aβ25-35 (100 μM) peptides were prepared through a 4-h incubation in PBS.

### Animals

All procedures met the guidelines of the U.K. Animals for Scientific Procedures and Directive 2010/63/EU of the European Parliament and the National Institutes of Health guide for the care and use of laboratory animals and were approved by the committee for the Care and Use of Laboratory Animals at Kochi University (permission number: L-00048) and followed ARRIVE guidelines 2.0. Fifteen male ICR mice (4 weeks old; Japan SLC, Hamamatsu, Japan) were housed per cage and maintained at controlled temperature (23 ± 1℃) and humidity (55 ± 2%) and a constant day-night rhythm (14/10-h light/dark cycle; lights on at 05:00) with free access to water and food. The experiment was conducted with a total of 15 mice.

### Intraventricular injection of Aβ25-35 and GSGFK

**Fig. 1:**
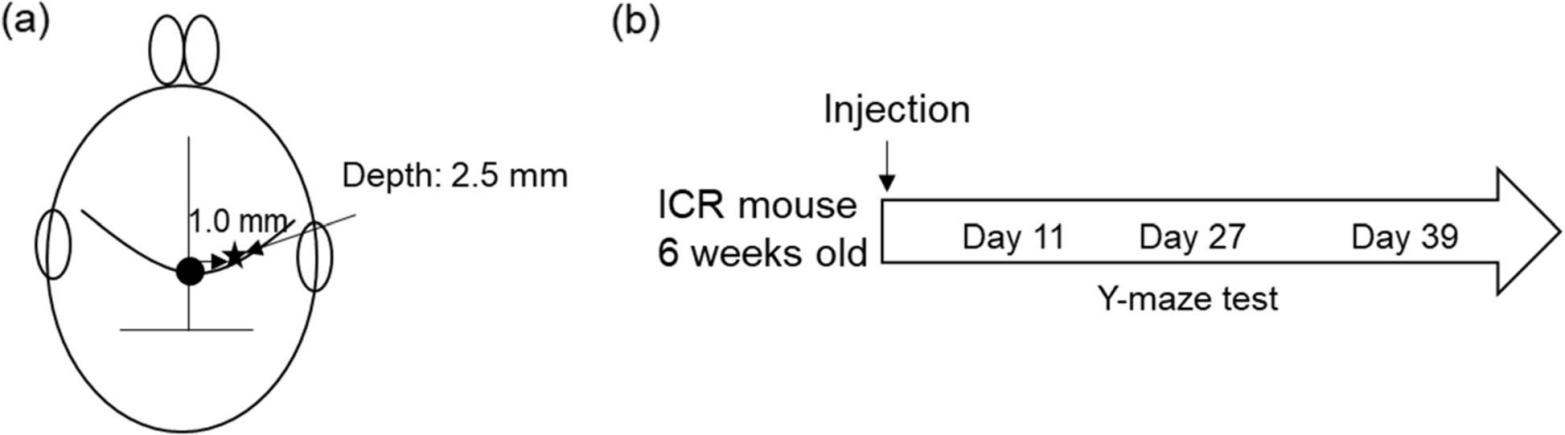
Methodology of animal experimentation.** a** Intraventricular injection of ICR mouse. **b** Experimental scheme

ICR mice were anesthetized with 1–3% isoflurane in a 75:25 mixture of nitrous oxide and oxygen. Mice were administered a stereotaxic injection of saline, 9 nmol Aβ25-35, or a mixture of 9 nmol Aβ25-35 and 9 nmol GSGFK in the right lateral ventricle (anteroposterior, 0.2; mediolateral, 1.0; dorsoventral, 2.0 mm; from the bregma and cortical surface) using a 10-μL Hamilton syringe (Fig. 1a; [29]).

### Spontaneous alternation in Y-maze test

The behaviour of mice in a spontaneous alternation Y-maze (40 cm long, three arms positioned at equal angles) was observed to measure short-term spatial memory deficits. Mice were placed at the end of one arm and allowed to explore freely during a 10-min session while the series of arm entries was recorded. Alternation was said to occur if a mouse entered an arm distinct from the two entered previously [28]. The percentage of relative alternation was calculated as [number of alternations / (number of total arm entries-2)] × 100. The time course of the Y-maze test is described in Fig. 1b.

### Phagocytosis assay

Microglial cell line BV-2 was maintained in Dulbecco’s modified Eagle medium (DMEM), supplemented with 5% foetal bovine serum in a CO_2_ incubator. The BV-2 cells were plated on 24-well plates (4 × 10^4^ cells/well) and incubated for 48 h. The medium was then replaced with serum-free DMEM, and the cells were treated with 0.01, 0.05, 0.1, 0.2, or 0.5 mM of GSGFK. After incubation for 24 h, the cells were further incubated with 1 μm of yellow-green carboxylate latex beads (Polysciences, Warrington, PA, USA; 1:5000 dilution) for 30 min in a CO_2_ incubator. Next, the cells were fixed with 4% paraformaldehyde for 20 min. Bead uptake was evaluated in three randomly selected fields from four wells in each experiment [30] using a laser confocal microscope and Cytation 5.

### Statistical analysis

All data are expressed as mean ± standard error of the mean. Statistical significance of the difference among experimental groups was measured using BellCurve for Excel (Social Survey Research Information Co., Ltd., Tokyo, Japan) followed by the Student’s *t*-test. The difference was considered significant at a *p*-value of 0.05. Graphs for the animal experiment were drawn in Graph Pad Prism [version 9.5.1 (528)] (Graph Pad Software, LLC).

## Results

### Screening of effective peptides

**Fig. 2:**
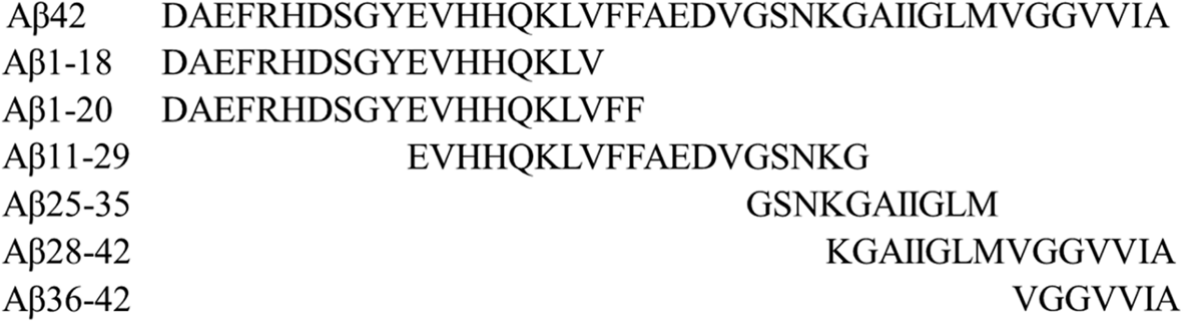
Amino acid sequences of Aβ42 and six kinds of amyloid-β fragment peptides

Aβ25-35 is an essential domain of Aβ42 aggregation [16, 17]. The ThT assay and electron micrographs showed that the ThT fluorescence intensity of Aβ25-35 correlated with fibril formation [5]. We previously reported that Catalytides cleave Aβ1-18, Aβ1-20, Aβ11-29, and Aβ28-42 [20, 21, 26, 31]. However, there are no reports of the aggregation potency of these peptides; thus, we compared the fluorescence intensity of six kinds of amyloid-β fragment peptides, including Aβ25-35, using ThT assay (Fig. 2).

**Fig. 3:**
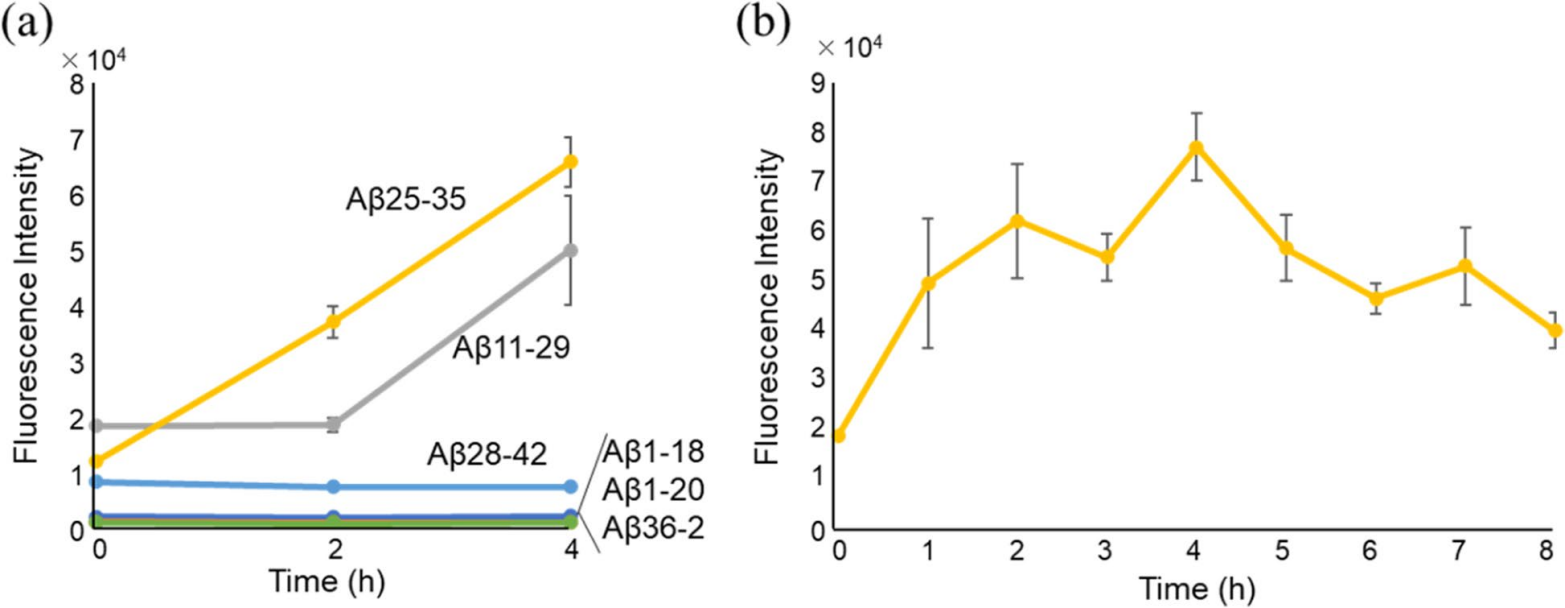
Thioflavin T-fluorescence profile of amyloid-β fragment peptides (Aβ-FPs).** a** Six kinds of 100 μM Aβ-FPs (Aβ1-18, Aβ1-18, Aβ11-29, Aβ25-35, Aβ28-42, and Aβ36-42) were incubated in Tris–HCl buffer (pH 7.5). The fluorescence intensity was measured at 0, 2, and 4 h. **b** Time course aggregation of Aβ25-35; 100 μM Aβ25-35 was incubated in Tris–HCl buffer (pH 7.5) and the fluorescence intensity was measured up to 8 h

When observed at 4 h, the fluorescence intensity of Aβ11-29 and Aβ25-35 solutions was increased. Among these two peptides, Aβ25-35 showed a higher aggregation potency (Fig. 3a), while the other peptides, Aβ1-20, Aβ1-18, Aβ28-42, and Aβ36-42, did not show any change. Next, we analysed Aβ25-35 aggregate formation for up to 8 h using the ThT assay in order to confirm the aggregation potency. The fluorescence intensity increased for up to 4 h and then decreased in a time-dependent manner, indicating that a 4-h incubation time was sufficient to monitor Aβ25-35 aggregate formation (Fig. 3b). Subsequently, we decided to use Aβ25-35 as a target peptide for the following experiments.

**Fig. 4:**
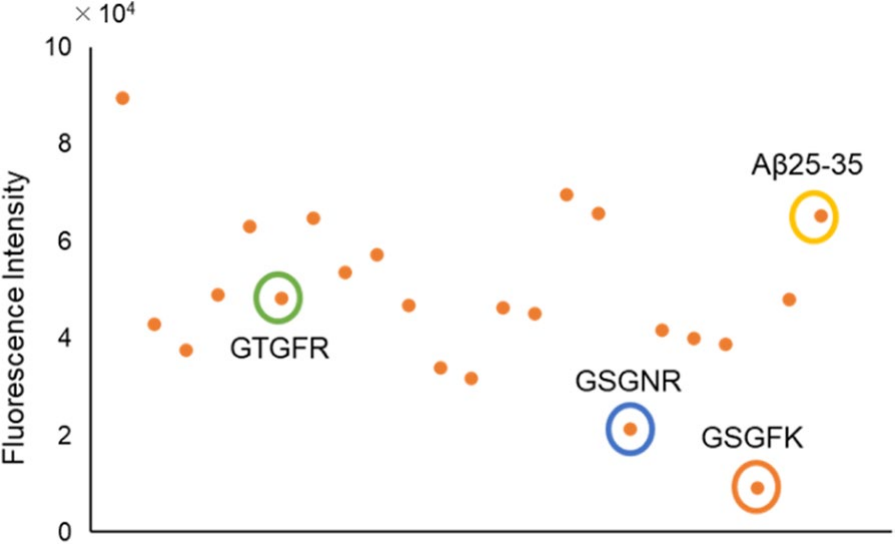
Screening for peptide that suppresses the aggregation of Aβ25-35 using Thioflavin T assay. The fluorescence intensity was measured after reaction with a mixture of each peptide (100 μM) at 37℃

We next screened the effect of 22 kinds of 5-mer peptide derivatives corresponding to GSGFR in the Box A region of Tob1 proteins against Aβ25-35 aggregation using the ThT assay. The fluorescence intensity of Aβ25-35 was calculated after a 4-h incubation. Among the 22 kinds of peptides, GSGFK and GSGNR notably reduced the fluorescence intensity (Fig. 4). These results suggested that both GSGFK and GSGNR may have an inhibitory effect on Aβ25-35 aggregation.

### Inhibitory effects on Aβ25-35 aggregate formation

**Fig. 5:**
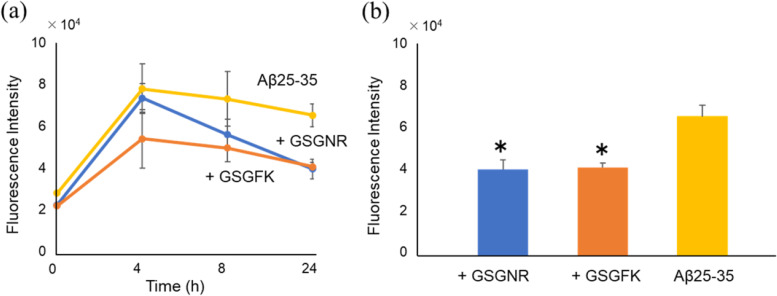
Thioflavin T-based evaluation of the inhibitory effect of GSGFK and GSGNR on Aβ25-35 aggregation.** a** Time course of the fluorescence intensity of each reaction mixture. Aβ25-35 (100 μM) was incubated with GSGNR (100 μM) or GSGFK (100 μM) at 37℃ for up to 24 h. **b** The fluorescence intensity after incubation of 24 h. Data shown are mean ± standard error of the mean (*n* = 3), versus Aβ25-35 group. *p < 0.05. GSGNR, *p* = 0.011; GSGFK, *p* = 0.007

To confirm the suppression of aggregation, we analysed the effects of GSGFK and GSGNR against Aβ25-35 using a triplicate assay for up to 24 h (Fig. 5a). GSGFK suppressed the aggregation of Aβ25-35 at 4 h. The fluorescence intensity plateaued after the 4-h incubation, and the pattern was similar to that of Aβ25-35 alone. On the other hand, GSGNR did not suppress the aggregation of Aβ25-35 at 4 h, but the fluorescence intensity was rapidly reduced between 4 and 8 h post-incubation. After 24 h, both peptides suppressed the fluorescence intensity at the same level and showed a significant difference as per the Student’s *t*-test (GSGNR: *p* = 0.011, GSGFK: *p* = 0.007). These data indicated that both GSGFK and GSGNR can suppress the aggregation of Aβ25-35 (Fig. 5b).

### Resolving effects on aggregated Aβ 25–35

**Fig. 6:**
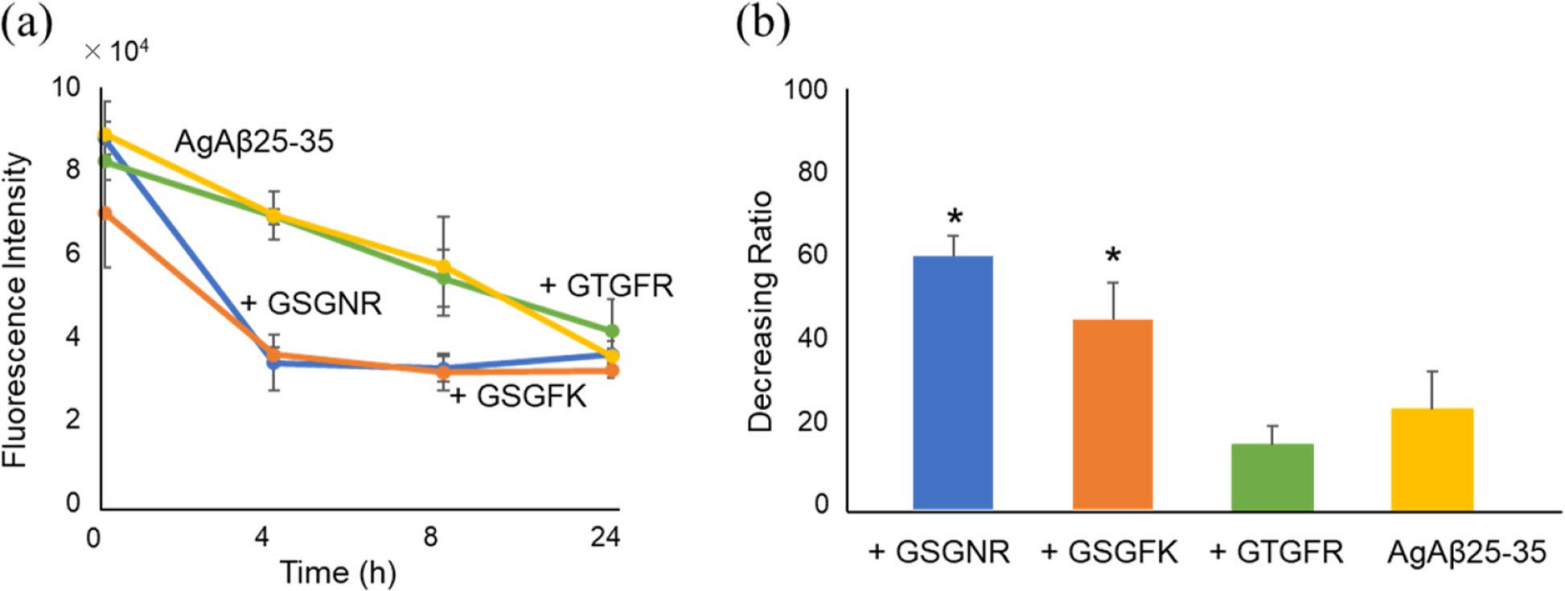
Resolving effects of GSGFK and GSGNR on aggregated Aβ25-35. Aβ25-35 (100 μM) was pre-incubated for 4 h to make its aggregated form, AgAβ25-35. **a** Time course of ThT-fluorescence intensity. **b** The decreasing ratio of the fluorescence intensity at 4 h. Decreasing ratio was calculated by measuring the fluorescence intensity at 0 and 4 h using the following formula: Decreasing ratio = fluorescence intensity at 0 h − fluorescence intensity at 4 h / fluorescence intensity at 0 h × 100. Data shown are mean ± standard error of the mean (*n* = 3), versus Aβ25-35 group. *p < 0.05. **a** GSGNR, *p* = 0.008; GSGFK, *p* = 0.002; **b** GSGNR, *p* = 0.014; GSGFK, *p* = 0.018

We analysed whether GSGFK and GSGNR, which suppressed the aggregation of Aβ25-35, can also resolve the Aβ25-35 that has already aggregated. First, Aβ25-35 was incubated at 37 °C for 4 h to allow the formation of Aβ25-35 aggregates (AgAβ25-35). AgAβ25-35 was co-incubated with GSGFK, GSGNR, or GTGFR. GTGFR which did not show inhibitory activity against the aggregation of Aβ25-35 was selected as a negative control (Fig. 4). The initial fluorescence intensity was higher due to the pre-incubation of Aβ25-35 (Fig. 6a). The intensities of AgAβ25-35 alone and AgAβ25-35 co-incubated with GTGFR decreased for 24 h in a time-dependent manner (Fig. 6a). The decrease in the fluorescence intensity of Aβ25-35 after reaching the maximum intensity value has also been reported and may be caused by the greater aggregative ability of β25-35 [16]. There was a reduction in AgAβ25-35 co-incubation with GSGFK or GSGNR. The level of the fluorescence intensity was significantly decreased (GSGFK: *p* = 0.002, GSGNR: *p* = 0.008) at 4 h and plateaued thereafter (Fig. 6a).

Since the initial fluorescence intensity was different for each reaction solution, we calculated the decreasing ratio based on the fluorescence intensity immediately after the start of the reaction and after 4 h. On comparison, both peptides showed significantly high decreasing ratios (GSGFK: *p* = 0.018, GSGNR: *p* = 0.014) (Fig. 6b). These results indicate that both GSGFK and GSGNR can resolve the aggregated Aβ 25–35.

### Animal experiments

**Fig. 7:**
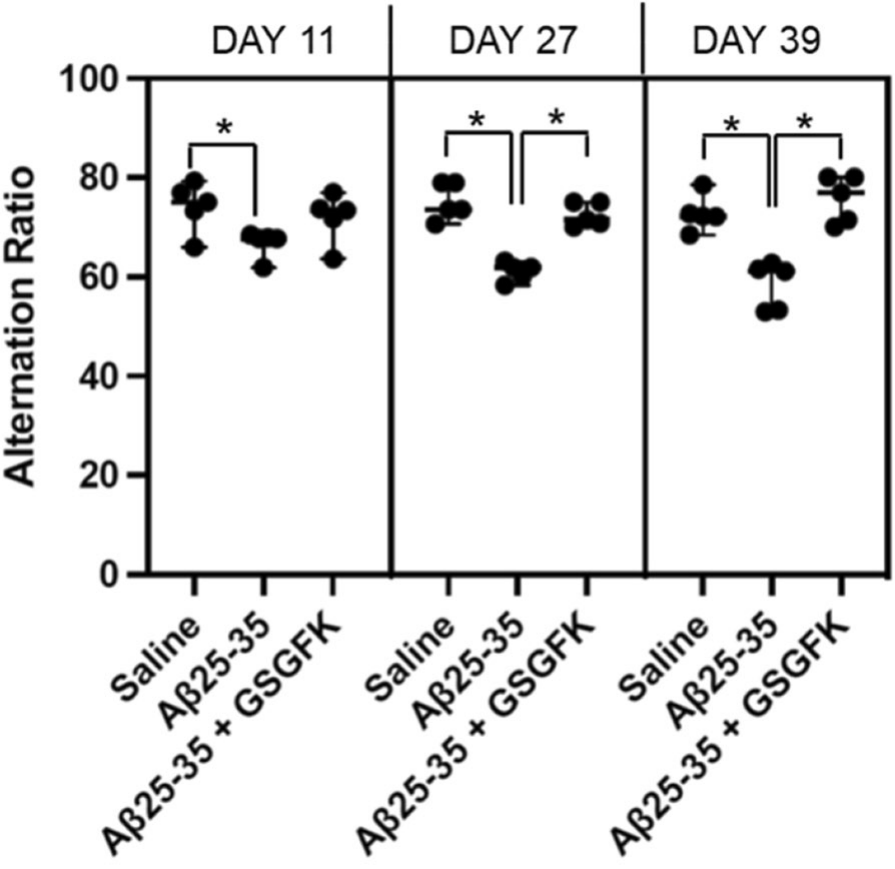
Prevention of short-term spatial-memory impairment by GSGFK. Y-maze test was performed at 11, 27, and 39 days after intracerebroventricular injection of saline, 9 nmol Aβ25-35, or a reaction mixture of 9 nmol Aβ25-35 and 9 nmol GSGFK. Data shown are mean ± standard error of the mean (*n* = 5). *p < 0.05. Saline: *p* = 0.021 (Day 11), *p* =  < 0.001 (Day 27), *p* = 0.001 (Day 39); GSGFK: *p* =  < 0.001 (Day 27), *p* = 0.001 (Day 39)

We investigated whether 5-mer peptides prevented the short-term spatial memory deficits induced by Aβ25-35 i.c.v. injection. Among the two types of 5-mer peptides, GSGFK, which showed an aggregation inhibitory effect, was selected. The Y-maze test was performed at 11, 27, and 39 days after injection to evaluate the inhibitory effects of GSGFK on aggregates by measuring the spontaneous alternation rate [28]. The alternation rate of the Aβ25-35-injected group induced significant short-term memory deficits as compared with that of the saline-injected group on all days (Day 11: *p* = 0.021, Day 27: *p* =  < 0.001, Day 39: *p* = 0.001). On the other hand, the Aβ25-35-co-injected GSGFK group showed a significantly heightened alternation rate compared with that of the Aβ25-35 group at 27 (*p* =  < 0.001) and 39 days (*p* = 0.001) (Fig. 7 and Table 1). These results indicated that GSGFK prevents the deficits in short-term memory induced by Aβ25-35.

**Table 1:**
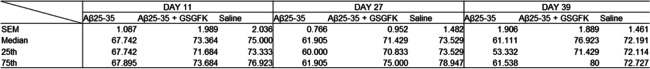
Standard error of the mean (SEM), median and 25th and 75th percentile alternation ratio

### Cell experiments

**Fig. 8:**
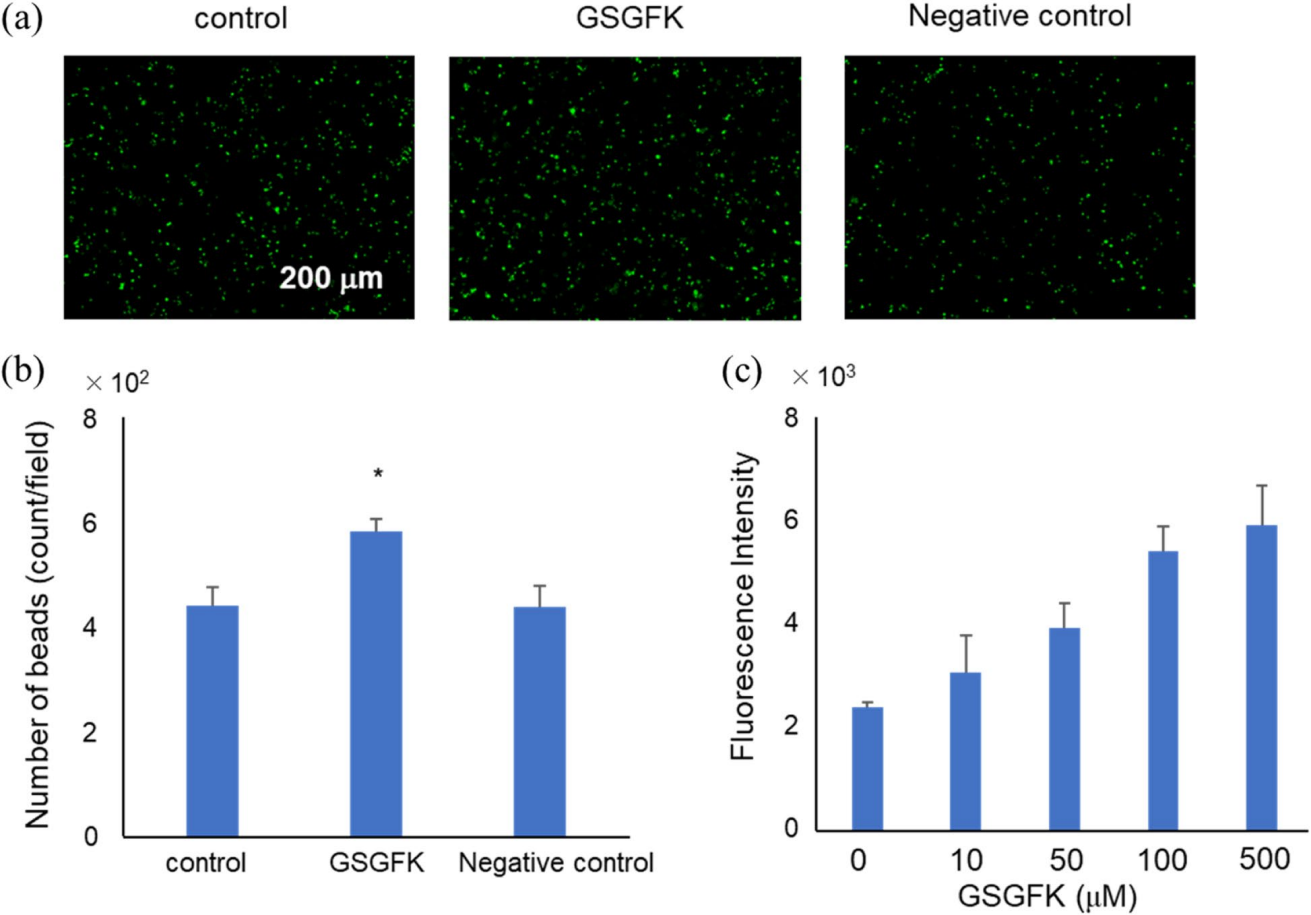
Effect of GSGFK on the phagocytic activity of BV-2 cells (BV-2 cells).** a** Representative image of fluorescent latex beads (green). **b** The amount of bead uptake in BV-2 cells. **c** BV-2 cells treated with PBS or GSGFK (500, 100, 50, and 10 μM). Excitation: 445 nm, emission: 500 nm. Data shown are mean ± standard error of the mean (*n* = 4). *p < 0.05. GSGFK, *p* = 0.028

We analysed the effect of GSGFK on the phagocytic activity of BV-2 cells. BV-2 cells were treated with GSGFK prior to the administration of yellow-green carboxylate beads. As shown in Fig. 8a and b, the bead uptake in BV-2 cells treated with 200 µM GSGFK was significantly higher (*p* = 0.028) than that of the non-treated (control) and 5-mer peptide cells, which did not show any inhibitory activity against Aβ25-35 aggregation (negative control). In addition, BV-2 cells were treated with 10–500 μM GSGFK prior to the administration of yellow-green carboxylate beads. As a result, the bead uptake was increased when treated with GSGFK in a dose-dependent manner (Fig. 8c). These results revealed that GSGFK activates the phagocytic ability of microglia to remove waste products in the brain.

## Discussion

Many trials for the development of AD treatment drugs have been conducted for a long period; however, the results have not been encouraging [7, 11, 32–36]. A few Aβ antibodies and vaccines aimed at inhibiting Aβ aggregation are still under development [4, 35, 36]. Recently, aducanumab, an antibody drug that inhibits Aβ aggregation, was approved by the U.S. Food and Drug Administration [7]. Aducanumab has been shown to inhibit the clinical manifestation of mild AD, but no drugs have yet been developed to radically eliminate Aβ and improve symptoms. Lecanemab is the latest Aβ antibody expected to be a more effective treatment for AD than aducanumab with a similar mechanism. These drugs are potentially effective in the early stages of AD. In the present study, we found that GSGFK and GSGNR are not only effective against aggregate formation of Aβ25-35 but also the aggregated Aβ25-35 already formed. In addition, GSGFK suppressed the cognitive deficits of the AD model mouse induced by Aβ25-35 injection.

Microglia are glia cells in the brain and spinal cord that maintain homeostasis by scanning the central nervous system. Microglia mediate inflammation and phagocytosis. Phagocytosis by microglia regulates brain development by controlling the neuronal population. In AD, phagocytosis is important for eliminating aggregated proteins such as Aβ [37, 38]. Recently, the relationship between microglia phagocytosis and age-related neurodegenerative disorders was studied [39, 40]. However, the role of microglia in AD remains controversial. Activated microglia release cytotoxic cytokines, which are considered to progress AD. In contrast, phagocytosis has been shown to decrease the progression of AD [41–43]. The timing of this shift in microglia from being protective to pathogenic remains unclear, but microglia have been shown to play neuroprotective roles during the early stage of AD through the promotion of Aβ clearance [44]. Thus, microglia are expected to be important for developing new therapeutic approaches for AD treatment. The current study revealed that GSGFK could play a neuroprotective role in the early stage of AD by inhibiting Aβ aggregation and promoting phagocytosis.

The development cost and side effects of antibody drugs, such as aducanumab and lecanemab, are considered problems; however, 5-mer peptides have no such limitations due to their weak side effects and lower manufacturing cost.

## Limitations

This study has some limitations. First, we investigated the effects of 5-mer peptides on Aβ25-35—an essential Aβ42 aggregation domain; however, the effects against Aβ42 aggregation were not studied. Next, we conducted only the Y-maze test to determine the effect of GSGFK on the AD model mouse. Other tests evaluating the cognitive state are required to validate the effects of 5-mer peptides observed in our study.

## Conclusion

**Fig. 9:**
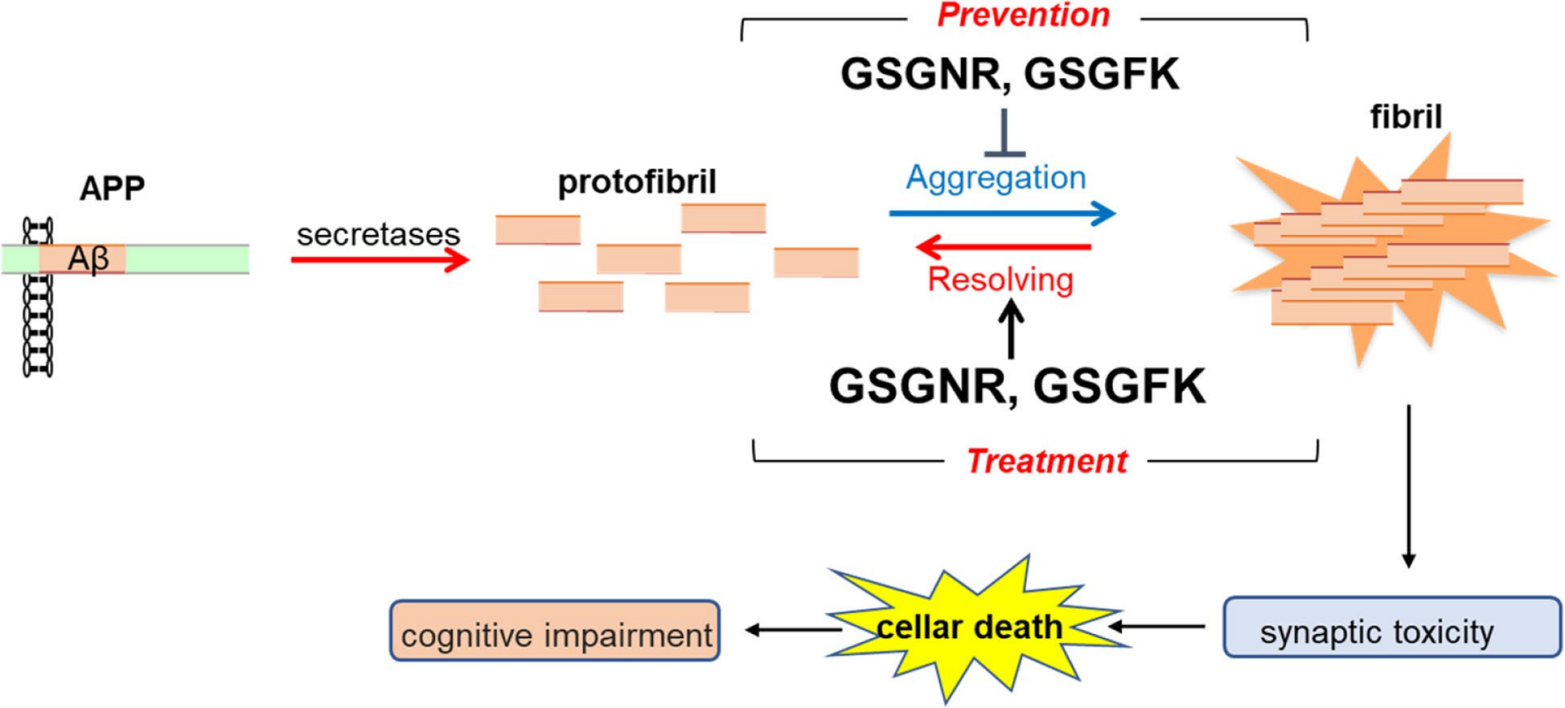
Effect of 5-mer peptides on protofibril and fibril in Aβ25-35

It is necessary to confirm the inhibitory effects of 5-mer peptides on Aβ42 or Aβ40 through animal experimentation using APP-knock-in and/or Aβ25-35-induced AD model mouse. Nevertheless, we believe that the 5-mer peptides GSGFK and GSGNR can resolve fibrils in addition to the inhibition of further fibril formation from protofibrils. In conclusion, 5-mer peptides are potential candidates for the prevention and treatment of AD (Fig. 9).

## Data Availability

The data and materials in this article are available from the corresponding authors on reasonable request.

## Abbreviations

Aβ: Amyloid beta
AD: Alzheimer’s disease
Aβ-FPs: Amyloid-β fragment peptides
APP: Amyloid precursor protein
DMEM: Dulbecco’s modified Eagle medium
ThT: Thioflavin-T
